# Feeding Practices, Maternal and Neonatal Outcomes in Vaginal Birth after Cesarean and Elective Repeat Cesarean Delivery

**DOI:** 10.3390/ijerph19137696

**Published:** 2022-06-23

**Authors:** Patryk Rudzinski, Inga Lopuszynska, Katarzyna Pieniak, Daria Stelmach, Joanna Kacperczyk-Bartnik, Ewa Romejko-Wolniewicz

**Affiliations:** 1 Students’ Scientific Group Affiliated to II Department of Obstetrics and Gynecology, Medical University of Warsaw, 02-091 Warszawa, Poland; ingalopuszynska@gmail.com (I.L.); katarzyna.m.pieniak@gmail.com (K.P.); daria.stelmach97@gmail.com (D.S.); 2 II Department of Obstetrics and Gynecology, Medical University of Warsaw, 02-091 Warszawa, Poland; joanna.kacperczyk-bartnik@wum.edu.pl (J.K.-B.); eromejkowolniewicz@wum.edu.pl (E.R.-W.)

**Keywords:** pregnancy, pregnancy outcome, repeat cesarean section, vaginal birth after cesarean, cesarean section, feeding method

## Abstract

Cesarean section rates are constantly rising, and the number of women with a prior cesarean considering a delivery mode for their next labor is increasing. We aimed to compare maternal and neonatal outcomes and feeding method in women undergoing vaginal birth after cesarean (VBAC) versus elective repeat cesarean delivery (ERCD). This was a retrospective cohort study of women with one prior cesarean delivery (CD) and no previous vaginal births, delivering vaginally or by a CD in a single institution between 2016 and 2018. 355 live singleton spontaneous vaginal and cesarean deliveries were included. 121 women delivered vaginally and 234 had a CD. Neonates born by a CD were more likely to have higher birth weight (*p* < 0.001), higher weight at discharge (*p* < 0.001), macrosomia (*p* = 0.030), lose >10% of their body mass (*p* = 0.001), be mixed-fed (*p* < 0.001), and be hospitalized longer (*p* < 0.001). Children born vaginally were more likely to be exclusively breastfed (*p* < 0.001). Women undergoing VBAC were more likely to deliver preterm (*p* = 0.006) and post-term (*p* < 0.001), present with PROM (*p* < 0.001), have greater PROM latency period (*p* < 0.001), and experience intrahepatic cholestasis of pregnancy (*p* = 0.029), postpartum anemia (*p* < 0.001), and peripartum blood loss >1 L (*p* = 0.049). The incidence of anemia during pregnancy was higher in the ERCD cohort (*p* = 0.047). Women undergoing VBAC are more likely to breastfeed their children, perhaps for the same reason they choose the vaginal method of delivery, as vaginal delivery and breastfeeding along with antibiotic use, are the most important factors decreasing the risk for future diseases in their offspring.

## 1. Introduction

Counselling women with a prior cesarean section remains a controversial topic in obstetric practice. The rate of vaginal birth after a prior cesarean delivery (VBAC) is on the decline worldwide [1,2]. On the other hand, the rate of cesarean section is steadily on the rise, and repeat cesarean section is the largest contributor to overall CD rates. As reported by the World Health Organization, in 2015, the rate of cesarean section in Robson group 5 (women with a previous cesarean delivery) ranged between 63.2% and 72.1% in low-income countries, 85.2% and 87.5% in middle-income countries, and 78.1% to 79.4% in high-income countries [3]. Additionally, the preference for a repeat cesarean delivery rises in the group of women after the prior cesarean section due to concerns about maternal and neonatal safety and morbidity [4,5,6]. Despite the fears, the ACOG Practice Bulletin number 205 deems vaginal birth after a previous cesarean a safe option for both a woman and a child, as it is linked to decreased maternal morbidity and a lower risk of complications in future pregnancies, also contributing to lowering of the overall CD rate at the population level [7,8]. Nevertheless, women and practitioners must bear in mind that many studies have shown that VBAC is associated with a higher uterine rupture rate [2,9,10,11,12,13,14,15].

Many prediction models for successful VBAC have been developed. When considering whether to opt for a VBAC or ERCD (elective repeat cesarean delivery), one must remember aspects contributing to the prognosis of success of either delivery. Some of these factors include maternal age at delivery, gestational age at delivery, estimated fetal body mass, and the time interval between deliveries.

This study aimed to examine the maternal outcomes, neonatal outcomes, and feeding method in women that underwent a vaginal birth after a caesarean and in women that chose an elective repeat cesarean delivery. The women were after a single prior cesarean delivery and nulliparous regarding vaginal births.

## 2. Materials and Methods

It was a retrospective cohort study comparing pregnancy course and maternal and fetal outcomes, as well as feeding method in singleton pregnancies after one prior caesarean section. Patient data were obtained from a hospital registry. The hospital’s database was searched for both maternal and neonatal records. We used the International Classification of Disease, 10th Edition (ICD-10) diagnostic and procedure codes and selected women who delivered live singleton pregnancies vaginally or by a cesarean section. The identified cohort was then reduced to fit our study criteria. The women were admitted and managed at a tertiary referral center in Warsaw, Poland. The hospital has an annual birth rate of approximately 3000 births/year. All pregnancies between 1 January 2018, and 31 December 2018 that resulted in elective repeat cesarean delivery were assessed. All pregnancies that resulted in vaginal birth after a caesarean delivered between 1 January 2016, and 31 December 2018 were assessed as well. Women classified as VBAC were those with spontaneous birth after only one prior cesarean delivery and no prior vaginal deliveries. Women classified as ERCD were those who had a cesarean delivery after only one prior cesarean delivery and no previous vaginal deliveries. Only women after one prior cesarean and no prior vaginal births that delivered live singleton were included in the study. Operative births, emergency cesarean deliveries, unsuccessful deliveries, or inductions of labor were excluded from the study. During the data collection period, the recommendations of the Polish Gynecological Society allowed women after a prior cesarean section to opt for a repeat cesarean section when she did not sign a form for TOLAC (trial of labor after a cesarean) [16]. Altogether, 234 women in the ERCD group and 121 women in the VBAC group were enrolled. Analyzed factors included maternal characteristics, maternal ante- and postpartum morbidities, neonatal characteristics and morbidities, management, outcome, and feeding practices.

Continuous variables were calculated as mean ± standard deviation, whereas categorical variables were calculated as a rate (%). Statistical significance for discrete data was determined by chi-squared test of independence and Cramér’s V was used to measure the association strength. Multivariate analysis of variance (MANOVA) was used to evaluate continuous data without missing values. Two variables (neonatal weight at discharge and time from previous cesarean section) had missing values; hence, we performed ANOVA for those datasets to avoid reducing the population size for other variables. The size of the missing data groups was as follows: for neonatal weight at discharge, 121 VBAC and 233 ERCD; for the time from previous cesarean, 80 VBAC and 128 ERCD. Statistical significance was defined as *p* < 0.05. For the calculations, IBM SPSS Statistics 26.0 was used.

The Bioethical Committee of the Medical University of Warsaw was informed about the study and has given a positive statement.

## 3. Results

### 3.1. Maternal Characteristics and Results

The total number of women included in the study was 355. Mean maternal age at delivery was similar in both groups (Table 1). Mean gestational age at delivery was similar in the VBAC and ERCD groups, but women undergoing VBAC were more likely to deliver preterm (*p* = 0.006) and at 40 or more weeks (*p* < 0.001). Time from a previous cesarean delivery was longer in the ERCD group; however, it was statistically insignificant.

Mean hospitalization duration was similar in the groups.

The incidences of diabetes mellitus (gestational DM type 1 and 2, pre-gestational DM), pregnancy-induced hypertension, pre-pregnancy hypertension, hypothyroidism, infection of the reproductive tract, cervical insufficiency, smoking, and postpartum blood transfusion were statistically insignificant. The incidence of anemia during pregnancy was significantly higher in the ERCD cohort (*p* = 0.047). More common in the VBAC group were intrahepatic cholestasis of pregnancy (*p* = 0.029), peripartum blood loss >1 L (*p* = 0.049), and postpartum anemia (*p* < 0.001). The differences in the rates of positive cervical culture and meconium-stained amniotic fluid were statistically insignificant. In the ERCD cohort, 2 amniotic fluid cultures were positive. Women undergoing VBAC were more likely to present with a premature rupture of membranes (*p* < 0.001) and have a longer PROM latency period (*p* < 0.001). The Cramér’s V of 0.764 for PROM incidence and degrees of freedom = 1 indicates a very large association between birth mode and PROM. For the rest of statistically significant variables, both categorical and continuous, the Cramér’s V was between 0.1 and 0.3 (degrees of freedom = 1), and the partial η^2^ was between 0.01 and 0.06, denoting a small association between the birth mode and those variables.

In both cohorts, some women received antenatal steroids, but the difference was statistically insignificant.

### 3.2. Neonatal Characteristics and Outcomes

The neonates born by cesarean section were more likely to have higher birth weight (*p* < 0.001), higher weight at discharge (*p* < 0.001), and be macrosomic (*p* = 0.030) (Table 2). They were also more likely to lose > 10% of their body mass (*p* = 0.001).

The incidence of cardiac complications, polycythemia, hypoglycemia, lenticulostriated vasculopathy, intrauterine growth retardation, low birth weight was similar in the neonates in the two cohorts. No statistically significant differences were observed in the neonates in respiratory complications and admission to the Neonatal Pathology Unit or Neonatal Intensive Care Unit (NICU). The neonates born to mothers from the ERCD cohort were more likely to require longer hospitalization (*p* < 0.001).

For the statistically significant variables, both categorical and continuous, the Cramér’s V was between 0.1 and 0.3 (degrees of freedom = 1) and the partial η^2^ was between 0.01 and 0.06, indicating a small association between the birth mode and neonatal characteristics and outcomes.

### 3.3. Feeding Method

The neonates born to mothers from the ERCD cohort were more likely to be fed with a combination of breastmilk and formula (*p* < 0.001) (Table 3). On the other hand, neonates from the maternal VBAC cohort were more likely to be breastfed only (*p* < 0.001). There was no statistically significant difference in the neonates being fed with formula only.

For the statistically significant variables, the Cramér’s V was between 0.1 and 0.3 (degrees of freedom = 1), meaning a small association between the birth mode and those feeding methods.

## 4. Discussion

Only a few studies have examined the outcomes in women whose characteristics are only one prior cesarean and no prior vaginal deliveries [13,17,18]. Many studies have had more liberal inclusion criteria and included women that have had one caesarean section, but also a varying number of previous vaginal deliveries [1,6,9,11,12,14,19,20,21]. We referred to both study types for comparison.

Our study shows that more pregnancies are delivered at the gestational age of <37 or ≥40 by women undergoing VBAC. This might be because the elective CD is usually scheduled for 39 weeks of gestation.

The time from a prior cesarean differed between the groups, with the ERCD having a longer interval between deliveries. It was on the borderline of statistical significance (*p* = 0.055), yet greater than the assumed statistical significance of the *p*-value. Another study showed no difference in the interdelivery interval [17].

Pregnancy complications, such as gestational and pregestational diabetes mellitus, pre-pregnancy and pregnancy-induced hypertension, hypothyroidism, infection of the reproductive tract, cervical insufficiency, and positive cervical culture, were statistically insignificant between the groups. This allows us to assume that both cohorts were relatively homogenous. The only difference was the incidence of intrahepatic cholestasis of pregnancy, which was higher in the VBAC group, and the incidence of anemia during pregnancy, which was approximately 6 times more prevalent in the ERCD group. Surprisingly, the rate of postpartum anemia was almost 2 times higher in the VBAC group.

Despite an almost 3-fold higher rate of postpartum blood transfusion in VBAC, the result was statistically insignificant. This is similar to the results of the study by Pont [21], in which the risk of transfusion was almost 4 times higher for the VBAC group than the ERCD group and statistically significant. Other studies also confirm that trend [9,11,18,22].

Peripartum blood loss >1 L occurred only in 2 women undergoing VBAC and no woman undergoing ERCD. The difference was found to be statistically significant in our study. Similar results were reported in other studies, showing that women delivering vaginally after a prior cesarean have statistically significantly higher rates of peripartum hemorrhage [13,14].

PROM incidence was much higher in women undergoing VBAC, as was the PROM latency period. This may be the reason why women ended up delivering vaginally as the labor begun spontaneously. The incidence in the ERCD may also be attributed to the fact that those deliveries were planned, hence the odds of this complication occurring were lower.

Children born from ERCD weighed 233.5 g more and were 173.2 g heavier when discharged, and both results are statistically significant. Some studies demonstrate opposite results [19].

Children born from ERCD were more frequently macrosomic than children born from VBAC (5.6% vs. 0.8%) and were more prompt to lose more than 10% of their birth weight (13.7% vs. 2.5%). Body weight loss >10% is defined in this paper as losing more than 10% of birth weight during the first 3 days of hospitalization, after which time period healthy newborns properly fed are discharged.

Cardiac, respiratory, and other complications were similar between the two groups of neonates in our study. Regarding respiratory outcomes, Lehmann, Yang, and Kok [10,13,19] demonstrated higher rates of infant respiratory distress syndrome (IRDS) in the ERCD group. Litwin [20] described a higher need for assisted ventilation in ERCD neonates, while Studsgaard [14] reported higher neonatal depression (defined in the paper as Apgar score 5 min after birth < 7 and/or pH < 7 in vessels of the umbilical cord) in the VBAC cohort.

Hospitalization associated with delivery was longer in the group of ERCD taking 5.6 days vs. 4.8 in the VBAC group.

Women who delivered vaginally after cesarean were more likely to breastfeed than those who delivered by ERCD. Mothers in both cohorts have gone through a prior cesarean delivery, and for the ERCD group, it was a second one. Nevertheless, ERCD mothers were still more likely to feed their children with breastmilk and formula. This is consistent with another study that looked at breastfeeding at birth and discharge in the same cohorts as in our study [9]. It showed that mothers delivering vaginally were more likely to breastfeed at birth and hospital discharge. In another study, lower rates of early initiation of breastfeeding, delayed initiation of breastfeeding, and non-exclusive breastfeeding have been associated with women undergoing cesarean delivery for the first time, compared with women delivering vaginally [23]. Possible reasons for these differences are: a longer recovery period after surgical procedure, pain, prolonged immobilization, or even diminished interest in the neonate due to the lack of initial skin-to-skin contact [24]. In the hospital where the data were collected, there is a policy of immediate skin-to-skin contact regardless of birth mode. Breastfeeding is always encouraged and instantly initiated. In spite of this adherence to UNICEF principles for breastfeeding promotion, there was, even if small in effect, an association between mode of birth and method of feeding.

NICU admissions were similar in both groups, with a percentage of 1.7% vs. 1.3%, respectively, for VBAC and ERCD. The ERCD group has higher NICU transfer according to Lehman and Studsgaard, while Fitzpatrick showed the opposite trend [9,14,19].

The strengths of our study include a coherent study group that predominantly consisted of Caucasian women, which eradicates ethnicity being a confounding factor. However, it can also be seen as a limitation as we are not sure if it applies to different ethnic groups. Our study focuses on a growing group of patients that are of clinical interest, as CD rates are on a continuous rise. Not many studies excluded patients with past vaginal delivery, which makes our study unique.

The limitations of our study include a small registry that hampers our capacity to assess differences in rare maternal and neonatal outcomes, as well as assess factors influencing feeding method more in depth, since there were only a few cases of formula-feeding only. The retrospective character of our study must also be considered. We only analyzed women who delivered by spontaneous vaginal birth and by an elective CD, not taking into account their intentions or trial of labor. No operative births, emergency cesarean deliveries, unsuccessful deliveries, or inductions of labor were analyzed or included in the study. This is important as induction of labor and augmentation of labor have been shown as aggravating factors of uterine rupture, postpartum hemorrhage, cesarean delivery, and NICU admissions [6].

Family planning must be taken into consideration while thinking about the next elective cesarean section, as more uterine scarification can cause more problems during future pregnancies. Moreover, we have to care about psychological aspects as well. Many women want to give birth vaginally to feel fulfilled and complete.

Both VBAC and ERCD have their risks; however, in both cases, they are relatively minimal.

While counselling, both the woman and practitioner must evaluate all contraindications, fears, and desires of an expectant mother to satisfy all needs of the mother and allow the mother-to-be to make an informed decision regarding her delivery course. Encouraging women to opt for VBAC may be beneficial not only because it reduces the overall CD rate, but also provides them with better management options for any future pregnancies.

## 5. Conclusions

This study shows that women undergoing VBAC were more likely to develop postpartum anemia and peripartum blood loss >1 L, while the ERCD cohort was more likely to have anemia during pregnancy.

It also shows that women from VBAC group had higher incidence of PROM, starting labor spontaneously and were more likely to deliver preterm and post-term.

Neonates of women from the ERCD group were larger, more likely to lose >10% of their birth weight and be hospitalized longer.

In both cohorts, the neonatal and maternal outcomes were rare (most of them accounting for less than 10% in the studied population). Our study supplies additional information useful to the process of decision-making regarding the delivery mode for practitioners and women who are after a prior cesarean.

Women undergoing VBAC are also more likely to breastfeed their children, perhaps for the same reason they choose the vaginal method of delivery, as vaginal delivery and breastfeeding, along with antibiotic use, are the most important factors decreasing the risk for future diseases in their offspring.

## Figures and Tables

**Table 1:** Maternal and pregnancy characteristics.

Variable ^1^	VBAC Group (*n* = 121)	ERCD Group (*n* = 234)	Pearson χ^2^	Cramér’s V	F-Value	Partial η^2^	*p*-Value
Maternal age at delivery [years]	33.3 (3.90)	33.5 (4.06)			0.21	0.001	0.645
Gestational age at delivery [weeks]	38.5 (1.58)	38.6 (0.96)			0.96	0.003	0.327
**Preterm birth < 37 weeks of gestation**	**13 (10.7%)**	**8 (3.4%)**	**7.70**	**0.147**			**0.006**
**Gestational age at delivery ≥ 40 weeks**	**33 (27.3%)**	**18 (7.7%)**	**24.86**	**0.265**			**<0.001**
Time from prior cesarean [years]	3.5 (2.13)	4.1 (2.39)			3.72	0.018	0.055
Hospitalization [days]	7.9 (10.85)	6.5 (3.44)			3.25	0.009	0.072
Diabetes mellitus	36 (29.8%)	73 (31.2%)	0.08	0.015			0.780
GDMG1	21 (17.4%)	35 (15.0%)	0.35	0.031			0.557
GDMG2	12 (9.9%)	27 (11.5%)	0.21	0.025			0.643
PGDM	3 (2.5%)	10 (4.3%)	0.73	0.045			0.394
PIH	3 (2.5%)	5 (2.1%)	0.04	0.011			0.837
Pre-pregnancy hypertension	5 (4.1%)	12 (5.1%)	0.17	0.022			0.677
Hypothyroidism	41 (33.9%)	69 (29.5%)	0.72	0.045			0.396
**Anemia**	**2 (1.7%)**	**15 (6.4%)**	**3.96**	**0.106**			**0.047**
Infection of the reproductive tract	4 (3.3%)	12 (5.1%)	0.62	0.042			0.433
Cervical insufficiency	0	3 (1.3%)	1.57	0.066			0.211
Smoking	3 (2.5%)	5 (2.1%)	0.04	0.011			0.837
**Intrahepatic cholestasis of pregnancy**	4 (3.3%)	1 (0.4%)	4.76	0.116			0.029
**Peripartum blood loss >1 L**	**2 (1.7%)**	**0**	**3.89**	**0.105**			**0.049**
**Postpartum anemia**	**70 (57.8%)**	**79 (33.8%)**	**19.01**	**0.231**			**<0.001**
Postpartum blood transfusion	**3 (2.5%)**	**2 (0.9%)**	**1.52**	**0.065**			**0.218**
Positive cervical culture	43 (35.5%)	87 (37.2%)	0.09	0.016			0.761
Positive amniotic fluid culture	-	2 (0.9%)					
Meconium-stained amniotic fluid	11 (9.1%)	11 (4.7%)	2.64	0.086			0.104
**PROM**	**104 (86.0%)**	**21 (9.0%)**	**207.16**	**0.764**			**<0.001**
**PROM latency period [hours]**	**6.2 (14.14)**	**1.1 (11.15)**			**13.62**	**0.037**	**<0.001**
Antenatal corticosteroids	6 (5.0%)	4 (1.7%)	3.08	0.093			0.079

^1^ Values presented as *n* (%) or mean (SD); statistically significant results were bolded.

**Table 2:** Neonatal characteristics and outcomes.

Variable ^1^	Children Born in VBAC Group (*n* = 121)	Children Born in ERCD Group (*n* = 234)	Pearson χ^2^	Cramér’s V	F-Value	Partial η^2^	*p*-Value
**Birth weight [g]**	**3273.9 (476.11)**	**3507.4 (454.08)**			**20.40**	**0.055**	**<0.001**
**Weight at discharge [g]**	**3130.7 (453.16)**	**3303.9 (428.01)**			**12.53**	**0.034**	**<0.001**
**Macrosomia**	**1 (0.8%)**	**13 (5.6%)**	**4.71**	**0.115**			**0.030**
IUGR	1 (0.8%)	1 (0.4%)	0.23	0.025			0.634
Low birth weight	3 (2.5%)	2 (0.9%)	1.52	0.065			0.218
**>10% loss of body mass**	**3 (2.5%)**	**32 (13.7%)**	**11.25**	**0.178**			**0.001**
Respiratory complications							
Pneumothorax	0	2 (0.9%)	1.04	0.054			0.308
Transient tachypnea of the newborn	7 (5.8%)	21 (9.0%)	1.12	0.056			0.291
nCPAP	7 (5.8%)	19 (8.1%)	0.64	0.042			0.424
Neopuff	3 (2.5%)	4 (1.7%)	0.25	0.026			0.621
Mechanical ventilation	0	5 (2.1%)	2.62	0.086			0.105
Other complications							
Cardiac complications	7 (5.8%)	14 (6.0%)	0.01	0.004			0.940
Hypoglycemia	2 (1.7%)	14 (6.0%)	3.47	0.099			0.062
Polycythemia	3 (2.5%)	3 (1.3%)	0.69	0.044			0.407
Lenticulostriated vasculopathy	0	2 (0.9%)	1.04	0.054			0.308
Hospitalization							
Neonatal Pathology Unit admission	24 (19.8%)	36 (15.4%)	1.13	0.056			0.289
NICU admission	2 (1.7%)	3 (1.3%)	0.08	0.015			0.779
**Hospitalization duration [days]**	**4.8 (2.58)**	**5.6 (1.88)**			**12.42**	**0.034**	**<0.001**

^1^ Values presented as *n* (%) or mean (SD); statistically significant results were bolded.

**Table 3:** Feeding methods.

Variable ^1^	Children Born in VBAC Group (*n* = 121)	Children Born in ERCD Group (*n* = 234)	Pearson χ^2^	Cramér’s V	*p*-Value
**Breastfed only**	**84 (69.4%)**	**111 (47.4%)**	**15.57**	**0.209**	**<0.001**
**Breastfed and formula-fed**	**35 (28.9%)**	**117 (50.0%)**	**14.49**	**0.202**	**<0.001**
Formula-fed only	2 (1.7%)	6 (2.6%)	0.30	0.029	0.583

^1^ Values presented as *n* (%) or mean (SD); statistically significant results are bolded.

## Data Availability

Not applicable.
